# Methods for controlled preparation and dosing of microplastic fragments in bioassays

**DOI:** 10.1038/s41598-023-32250-y

**Published:** 2023-03-30

**Authors:** Hayden Boettcher, Tobias Kukulka, Jonathan H. Cohen

**Affiliations:** 1 School of Marine Science and Policy, University of Delaware, Lewes, DE 19958 USA; 2 School of Marine Science and Policy, University of Delaware, Newark, DE 19971 USA

**Keywords:** Environmental impact, Marine biology

## Abstract

Microplastic fragments (microfragments) are among the most abundant microplastic shapes found in marine ecosystems throughout the world. Due to their limited commercial availability, microfragments are rarely used in laboratory experiments. Here a novel method of microfragment production has been developed and validated. Polyethylene and polypropylene plastic stock (2 and 3 mm thick respectively) was ground using a cryomill, washed, and rinsed through a stack of sieves. Microfragments were prepared at three distinct size classes (53–150, 150–300, 300–1000 μm) and were confirmed to be accurate and consistent in size. Employing a novel ice cap dosing technique, microfragments were accurately dosed into experimental vials while excluding headspace, facilitating particle suspension without the aid of chemical surfactants. A proof of principle ingestion experiment confirmed the bioavailability of 53–150 μm polyethylene microfragments to brine shrimp *Artemia* sp. Together, these methods provide a controlled way to produce and dose microplastic fragments for experimental and analytical research.

## Introduction

With society's increasing reliance on plastics, and rapid increase in production and ensuing disposal, the environmental and economic implications of plastic pollution are a global concern. Microplastics (mp; 1–5000 µm particles) are the most abundant form of plastic pollution in the marine environment and are found in a large variety of shapes including fragments, fibers, beads, and films^1^. Microplastic debris often begins as land-derived waste, entering estuaries and coastal waters largely through the mismanagement of coastal waste^2^. Terrestrial sources of microplastics are numerous, including wastewater effluents^3,4^, landfills^5^, synthetic clothing^6,7^, tire wear^8,9^, and fishing gear^10^. Microplastics are ubiquitous in the marine environment from coastal waters and estuaries^11,12^, to deep-sea sediments^13,14^. Due to their small size, microplastics are bioavailable to a wide range of marine organisms^15^. Effects may arise from physical interactions with the particles (e.g., ingestion, entanglement), exposure to chemical additives in the plastics^16^, or subjection to pathogens due to biofouling^17^. Evaluating the risks posed by microplastics is a key goal for environmental regulators and legislators^16,18^ as they are environmentally persistent pollutants that are only expected to increase in number over time^19,20^.

Fragments and fibers are among the most observed microplastic shapes in the marine environment^11,21–23^. Typically created through the deterioration of larger macroplastic debris, microplastic fragments (microfragments) are irregularly shaped particles commonly composed of polyethylene, polypropylene and polystyrene^11,22,24^, though a wide range of additional types have been reported. Microfragments, and microplastics in general, are highly variable in size, with observed concentrations largely reliant on sampling location and technique. For example, microplastics sampled in the Delaware and Chesapeake Bays (USA) with 200–333 µm mesh nets reported microfragments ranging from 300 to 1000 µm at concentrations averaging between 0.19 and 1.24 pieces per cubic meter^11,22,23^. Additionally, water sampling conducted with similar nets throughout the UK Channel, North, and Celtic Sea found the majority (67%) of collected microplastics to be larger fragments ranging from 1000 to 2790 µm at concentrations of 0–1.5 pieces per cubic meter^25^. The persistence of microfragments is not just limited to coastal marine environments. Microfragments were also among the most common microplastic shapes sampled in lakes^26,27^, rivers^28–30^, and terrestrial sediments^31^ throughout the world. Furthermore, microplastic fragments have been found inside seabirds^32,33^, fish^34,35^, mussels^36,37^, and crustaceans^38,39^, illustrating their bioavailability once they enter the environment.

As the amount of research on microplastics has continued to increase, few knowledge gaps have become more glaring than the mismatch of plastics observed in the field and those used in the laboratory setting^40–42^. While microplastic fragments have been observed in over 20% of field studies, they were included in only 3% of laboratory studies, highlighting the divide between observational and experimental work^43^. Multiple studies have illustrated the significant impact that the size, shape, and polymer type of microplastics used in laboratory experiments can have on the results^44,45^. Therefore, it is important that researchers have the flexibility to select specific microplastic types for their experiments. Additionally, many previous studies coated microplastics in a thin layer of surfactant to achieve a homogenous distribution of particles in solution^46–48^. Surfactants can have toxic effects on aquatic organisms^49,50^and may also increase the proliferation of bacterial growth on the surface of microplastics^51^. Here, we introduce a novel ice cap dosing technique capable of producing accurate microplastic concentrations for experimental use. This technique removes headspace in the vial, preventing surface accumulation of the particles, and facilitating the suspension of the microplastics in solution without the aid of chemical surfactants.

The lack of microfragment use in previous studies can be attributed to (i) a lack of commercially available options and (ii) the absence of a standardized microfragment production procedure. Microplastic beads and pellets of a few common polymer types are available for purchase and are a convenient option. These beads have been successfully incorporated into many important toxicological studies^52–56^, prompting questions on how the results might change with more commonly observed microplastic shapes such as fragments. While fragments have been prepared and used in recent studies^44,57^, there currently lacks a consistent and reproducible method to produce and apply them. Previous work has highlighted the promise of cryomilling for creating microplastic fragments^58–60^; here, we introduce a comprehensive production protocol covering a wide range of microfragment sizes. Tewari et al.^58^ were successful in creating polypropylene and polyethylene microplastic fragments in the 2–125 μm size range using cryogenic grinding and sieving. Additional studies have used sieves to obtain specific size-fractions of microplastics^59,60^ though size distribution data on the resulting fragments was not published. The protocol detailed here builds on the previously published cryomill/grinding techniques by incorporating washing steps into the process, an important addition that facilitates the removal of ultra-fine microplastic contamination from the target size classes. Mention of this contamination was notably absent from each of the previously published cryomill methods^58–60^ and was a persistent issue until the washing steps were introduced. Additionally, the use of an interchangeable sieve stack affords this microfragment production method the ability to create multiple size classes of fragments in a single production run and the flexibility to choose specific size ranges.

Previously published microplastic production methods such as the microfiber production technique published by Cole^61^ opened the door for researchers to employ standardized microplastic fibers in their own work^44,62–64^. Likewise, the method presented here provides a standardized microplastic fragment production procedure. Microfragments were created through grinding in a cryomill, washed with a tween solution and sieved into specific size classes. Fragments were imaged and analyzed in ImageJ to assess consistency in size distribution. Furthermore, a novel ice cap technique for dosing and suspension is tested and validated. Finally, a six-hour ingestion experiment with brine shrimp *Artemia* sp. was performed to assess the bioavailability of 53–150 μm polyethylene microfragments.

## Results/discussion

### Microfragment production

**Figure 1:**
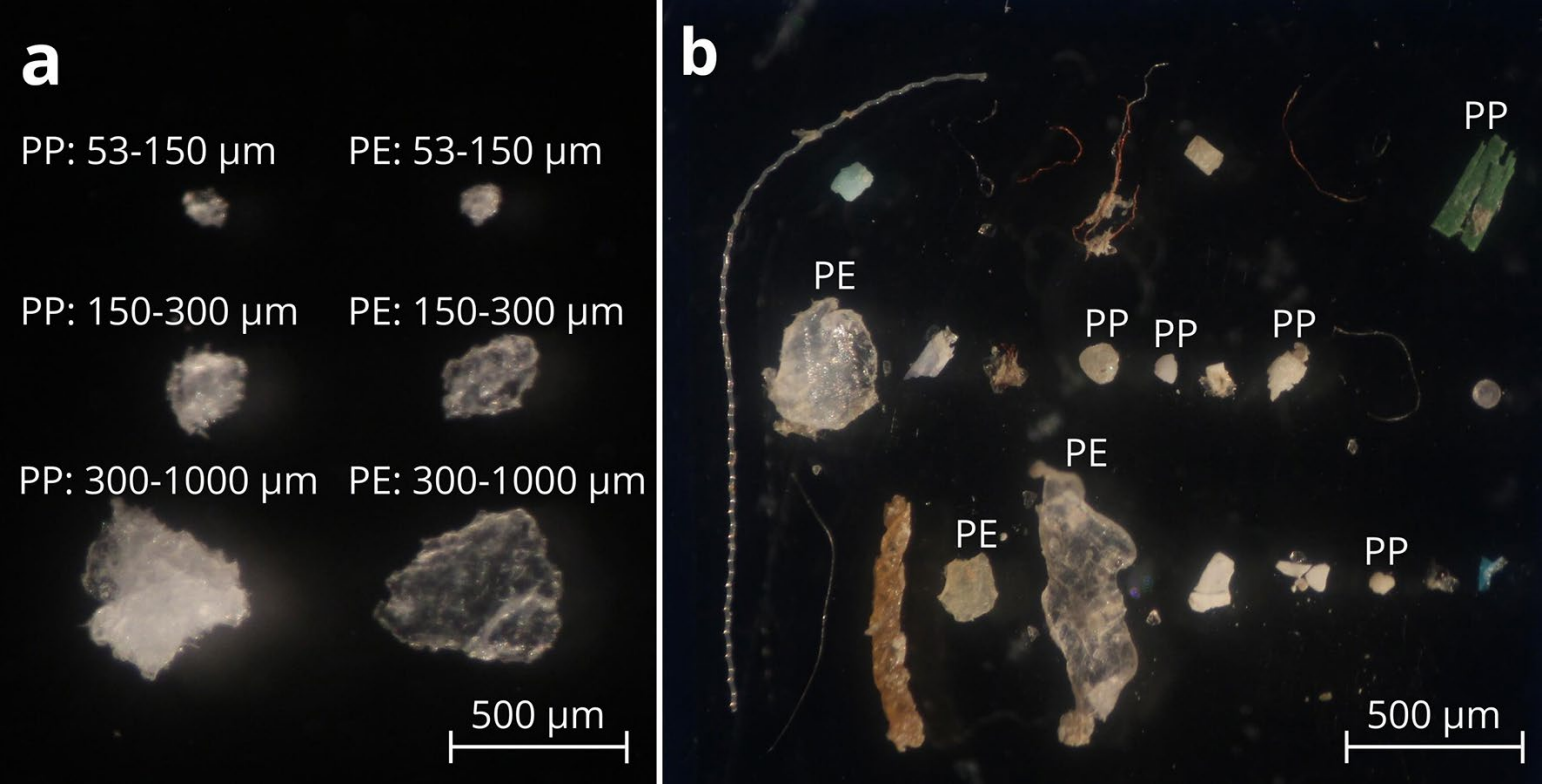
Manufactured and field sampled microplastic fragments. Micrographs: (**a**) Polypropylene (PP) and polyethylene (PE) microfragments from each size class produced using the microfragment production protocol; (**b**) Polypropylene and polyethylene microfragments sampled in the Delaware Bay. Field sample polymer types confirmed by micro-FTIR (Spotlight 200i micro-FTIR).

**Figure 2:**
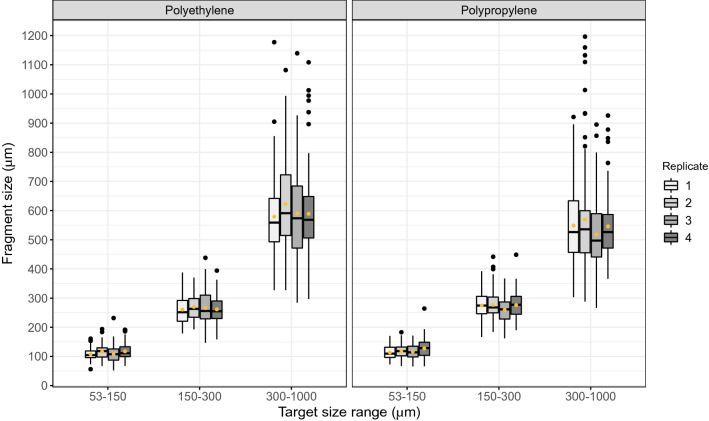
Polyethylene and polypropylene microfragments matched their targeted size ranges: 53–150, 150–300, and 300–1000 μm. Box-and-whisker plots illustrate the full spread of data for each polymer type, size class and production replicate including median, inter-quartile and min–max values. Mean microfragment size (yellow dots) fell within the target range for each sample. Size classes were all significantly distinct from one another (ANOVA, Tukey post-hoc test, *P* < 0.05).

**Figure 3:**
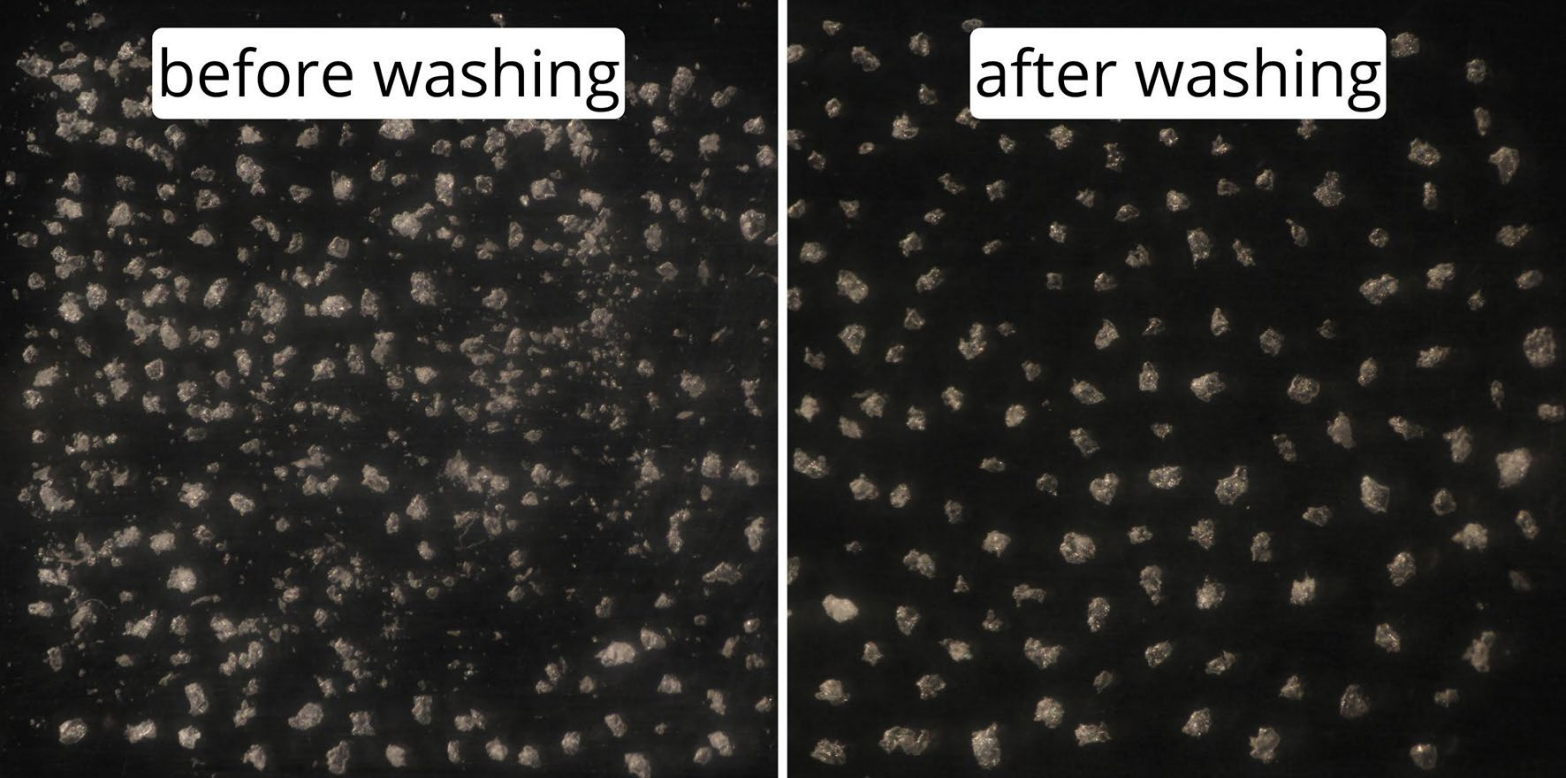
Polypropylene microfragments (150–300 μm) before and after washing in 0.1% Tween-80 solution. Microfragments were mixed at 600 rpm for ten minutes to separate ultra-fine microplastics that were contaminating the size fraction.

Employing a process of cryogenic grinding, washing, and sieving, the microfragment production protocol proved effective in creating polyethylene and polypropylene microplastic fragments of three controlled size classes (53–150, 150–300, and 300–1000 μm; Fig. 1). These fragments closely mimic the jagged, irregular nature of microplastic fragments collected in marine samples. Microfragment size distributions closely matched the target range, with the mean fragment size consistently falling within the target range (Fig. 2). Outliers with sizes greater than the targeted size class were occasionally observed and can be attributed to a variation in shape (i.e., fragments that are irregularly long and narrow). Significant differences in mean fragment size were occasionally observed between individual production replicates of the same size class (ANOVA, Tukey post-hoc test, *P* < 0.05). This shows that variability between production runs needs to be considered. This variation could be mitigated by combining production runs before size analysis and subsequent use in bioassays. Despite these differences, the mean and median fragment size always fell within the target range for all replicates. Should tighter size distributions be required, additional sieves could be added to the sieve stack step of the protocol. Key to the accuracy of this process was the addition of a washing step in 0.1% tween-80 solution. Washing and agitating the fragments before the final sieving step facilitated the removal of ultra-fine plastic contamination from each size class (Fig. 3). Without this step, contamination would cause inconsistencies in microfragment size as well as inaccurate concentrations in laboratory experiments. With the capacity to produce hundreds of thousands of microplastic fragments in two hours of work, this method will keep pace with the number of microplastics required for rigorous experimentation. The present study focused on polyethylene and polypropylene, both of which can be classified as crystalline/semi-crystalline thermoplastic polymers. We hypothesize that this production technique will work with other crystalline thermoplastics (e.g., nylon, polyester), though further testing will need to be conducted to assess the applicability of this technique with other plastic types.

### Microfragment dosing and bioavailability experiment

**Figure 4:**
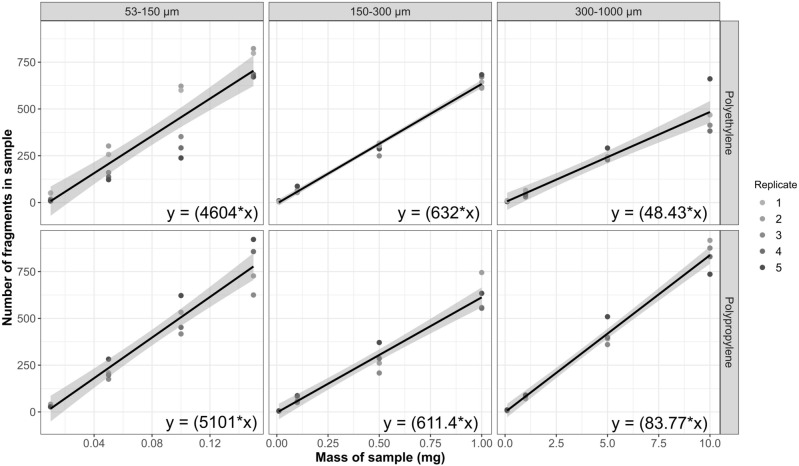
Linear regressions for each size class and polymer type. The origin for each regression was set through 0.0. Equations resulting from each regression (displayed in their respective panel) were used to calculate the amount of microplastics to add to an experimental solution for each target concentration (y = target number of microplastic fragments, x = mass of microplastics to be weighed out in mg). All regressions confirmed a significant linear relationship between the number of fragments and the mass of the sample in mg (*P* < 0.001, Supplementary Table S1).

The ice cap method proved to be an accurate and consistent method for dosing and suspending microplastic fragments into experimental solutions. Microfragments were frozen in a dome of ice, attached to the underside of a vial cap, and twisted onto the vial, releasing the fragments into solution. When testing the method with 150–300 μm polyethylene and polypropylene fragments all but one measured concentration fell directly within the target range (Table 1). The single group that did not fall within the target concentration was the polyethylene 100 mp/mL group, which was measured at a slightly higher concentration of 103 ± 1.27 mp/mL. The respective doses (i.e., mg of microfragments to be added) for each concentration were calculated using dosing equations. These equations are specific to each size class and polymer type and were obtained via linear regression (microfragment sample weight in mg ~ number of microfragments; Fig. 4). Due to their extremely small size, microplastics are often hydrophobic and difficult to incorporate into experimental solutions. If an air/water interface is present (e.g., air bubbles) microplastics will congregate around that surface. By removing the headspace for air in the experimental vials, the ice cap method proved to be effective in suspending microfragments in the experimental solution without the aid of chemical surfactants. Where this technique succeeds in accuracy and consistency, it lacks in speed and ease of preparation. From start to finish, preparing 10 vials of a target microfragment concentration takes around three hours. Compared to commonly used serial dilution methods^65,66^ the ice cap dosing method may take longer to set up but could provide more accurate and consistent microplastic concentrations.

**Table 1:** Validation of ice cap dosing concentrations.

Polymer type	Equation	Calculated mass (mg)	Nominal concentration (mp/mL)	Actual concentration (mp/mL, ± 1 sd)
Polyethylene	y = (632*x)	0.032	1	0.98 ± 0.03
		0.316	10	9.95 ± 0.05
		3.165	100	103.04 ± 1.27
Polypropylene	y = (611.4*x)	0.033	1	0.97 ± 0.03
		0.327	10	10.05 ± 0.09
		3.271	100	99.99 ± 1.66
Control	n/a	0	0	0.00 ± 0.00

Actual concentrations (n = 3) of polyethylene and polypropylene microfragments (150–300 μm) closely matched their nominal concentrations using the ice cap dosing method. Calculated masses for each nominal concentration were determined by linear regression dosing equations (see Fig. 4).

**Figure 5:**
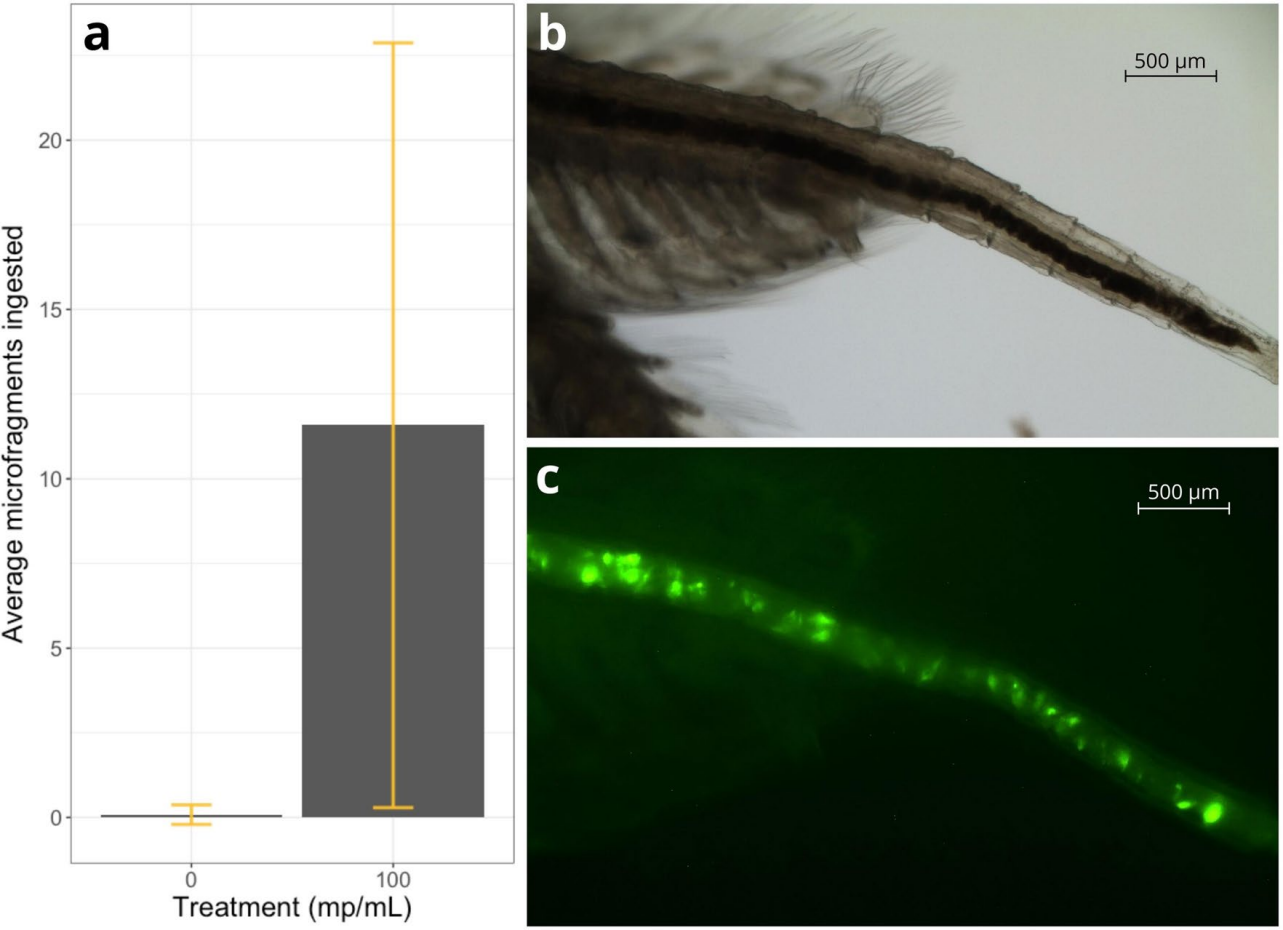
Polyethylene microfragments successfully incorporated into microplastic bioavailability experiment. (**a**) Average number of microfragments ingested per individual in the control (0 mp/mL, n = 12) and experimental (100 mp/mL, n = 24) treatments following the six-hour bioavailability experiment (± one standard deviation). (**b**) Natural light and (**c**) fluorescent micrographs of polyethylene microfragments (53–150 μm) inside the intestinal tract of an adult brine shrimp *Artemia* sp. with 520–542 μm fluorescent excitation (EVOS FL Auto).

A proof of principle experiment was conducted to assess the bioavailability of the produced microfragments. Brine shrimp *Artemia* sp. were exposed to control (0 mp/mL, n = 12) or microfragment (100 mp/mL, n = 24) treatments for six hours, after which ingestion and microplastic body burden were assessed. Results of the proof of principle experiment confirm the bioavailability of polyethylene fragments (53–150 μm) to brine shrimp (Fig. 5). Microplastic fragments were identified in the intestinal tract of 92% of tested *Artemia* sp. Individual plastic body burdens ranged from 0 to 48 fragments, with an average of 12 microfragments per organism. A single ingested microfragment was discovered in the control group. Microfragments were found from the stomach through the end of the intestinal tract, suggesting that they can move through the digestive system and are ultimately excreted. While *Artemia* sp. have become a useful test organism in assessing the bioavailability of microplastic beads and fibers^61,67^, this is the first indication that they can ingest microplastic fragments as well.

## Methods

### Plastic stock material

Microfragments were produced from sheets of 1 mm thick stock material. Plastic stock was selected to represent polymer types commonly found in marine samples—polyethylene (LDPE; Goodfellow ET313010), and polypropylene (PP; Goodfellow PP303100). Prior to processing, plastic sheets were cut into 5 × 5 mm squares to ensure a consistent starting size and shape.

### Microfragment production protocol

Microfragments were produced using a combination of cryogenic grinding and sieving/washing steps. Plastic stock (30 squares, ~ 5 g) was loaded into a cryomill (SPEX 6775 Freezer/Mill) and submerged in liquid nitrogen for a ten-minute cooling period. The sample was then ground for one minute and thirty seconds at an impact rate of 10 cps, followed by one minute of cooling. This cycle was repeated a total of four times for each sample. The resulting fragments were transferred to a stack of dry metal sieves (1000, 300, 150 and 53 μm; Hogentogler & Co.). The top sieve was sealed with parafilm, and the stack was shaken by hand for 5 min. The material on each sieve was rinsed into individual beakers with 0.2 μm-filtered deionized water (F-DIw) and vacuum filtered onto 20 μm polycarbonate filters. This process resulted in three size fractions: (i) 53–150 μm; (ii) 150–300 μm; (iii) 300–1000 μm. The microfragments on each filter were then poured into a respective glass beaker containing 100 mL of 0.1% (v/v) Tween-80/F-DIw solution. Using a stir plate and stir bar each sample was mixed at 600 rpm for ten minutes to suspend and separate smaller particles that may have been contaminating the size fraction. Each beaker was then poured back through its respective sieve and rinsed with F-DIw for five minutes. Microfragments collected on the sieve were vacuum filtered onto a 20 μm polycarbonate filter. The filter was transferred to an aluminum dish and enclosed inside a glass petri dish to dry for 24 h. While it was not tested, it may be possible to skip the initial dry-sieving step, and instead, start with the washing step. This could increase sample yield and save time.

### Microfragment analysis

To assess the consistency of the production technique, the entire production process was repeated four times for each polymer type (polyethylene and polypropylene). Subsamples of each replicate were spread onto individual glass slides (the mass of each subsample was relative to the size class). Microfragments were quantified by hand under a stereo microscope (Zeiss Stemi 2000-C) and photographed (Canon EOS Rebel T3i). A linear regression (number of fragments ~ subsample mass) was used to calculate a dosing equation for each polymer type and size class. Microfragment size was determined by measuring the longest axis (ImageJ, FIJI) of 100 fragments from each production replicate. Sample images were selected at random, and fragments were measured from left to right until the limit of 100 was reached. To confirm polymer integrity, analysis by micro-FTIR (Spotlight 200i micro-FTIR) was performed on subsamples of each polymer type. Furthermore, spectral comparisons of microfragments before and after the Tween-80 washing step were conducted to confirm a lack of residue on the produced fragments.

### Novel ice cap dosing protocol

Polyethylene and polypropylene microfragments were weighed onto 22 × 22 mm cover glass inside of a microbalance (Mettler UMT2). Once removed from the balance, a small dome of F-DIw was then pipetted onto the microfragments releasing them into the dome of water. Forceps were used to release any remaining microfragments from the cover glass into the surface of the dome. Microfragments that are not released into the surface of the dome may be left behind during the subsequent transfer to the vial cap. The cover glass was then transferred to a − 80 °C freezer for thirty minutes. Once frozen, the cover glass was picked up and briefly heated by fingertip to release the frozen dome. The dome was then transferred to the inside surface of the vial cap. Only black polypropylene vial caps with polytetrafluoroethylene (PTFE) linings were tested. Changes in the vial lining material may influence the effectiveness of this technique. The vial cap was placed back into the − 80 °C freezer for an additional thirty minutes. Prior to dosing, the experimental vials were overfilled with F-DIw, forming a convex dome of water at the mouth of the vial to ensure zero airspace for bubble formation. The cap was removed from the freezer, quickly twisted onto the vial, and inverted. The resulting vial was free from air pockets and as the ice melted, the microfragments were released and suspended in solution. While contamination was not assessed in the present study, we recommend that the pipetting be done in a laminar flow hood and prepared vial caps be stored in covered containers (e.g., petri dishes or covered trays) to avoid airborne contamination.

### Ice cap dosing analysis

An experiment was conducted to determine the accuracy and consistency of the ice cap dosing technique. Treatments consisted of (i) microfragment free controls; (ii) polyethylene microfragments (150–300 μm) and (iii) polypropylene microfragments (150–300 μm). Target concentrations for both plastic treatments included 1, 10, and 100 mp/mL. Each of the three treatments contained three replicates, resulting in 21 total vials. Ice caps were prepared following the described technique and screwed onto 20 mL glass scintillation vials. Vials were held on a rotating plankton wheel (6 rpm) for six hours. Control vials, as well as treatments targeting 1 and 10 mp/mL were vacuum filtered onto 0.20 μm polycarbonate filters and hand counted under a stereo microscope (Zeiss Stemi 2000-C). Treatments targeting 100 mp/mL were rinsed into glass beakers with F-DIw and diluted to 50 mL in a 5% (v/v) Tween-80/F-DIw solution. Samples were then stirred (400 rpm) for five minutes to evenly suspend microfragments in the solution. Three 1 mL subsamples were taken from each treatment and enumerated on a 1 mL gridded well slide (i.e., Sedgwick-Rafter chamber) under a compound microscope (40X, Olympus CX31). The average of these three counts was used to calculate the concentration of mp/mL for each replicate.

### Microfragment bioavailability experiment

To assess the applicability of the microfragments in laboratory experiments, a proof of principle experiment was conducted. For bioimaging purposes, microfragments were fluorescently labeled using Nile Red per the methods detailed by Cole (2016). Adult brine shrimp *Artemia* sp. were starved for six hours before being placed in 20 mL glass vials of F-DIw. Treatments consisted of: (i) microfragment free controls (n = 12) and (ii) fluorescently labeled polyethylene microfragments (53–150 μm) at 100 microfragments/mL (n = 24). Microfragment concentrations were achieved using the ice cap dosing method. To ensure a constant suspension of microfragments, vials were held on a rotating plankton wheel (6 rpm) in an environmental chamber (25 °C) for 6 h. Each vial was then emptied and rinsed into a 100 mL glass beaker. Artemia survival was noted, and individuals were then rinsed with F-DIw and transferred to individual wells containing 250 μL of 4% formaldehyde. Specimens were then transferred to well slides and visualized under a fluorescent-coupled microscope (EVOS FL Auto; RFP light cube, 520–542 μm excitation) where the proportion of individuals containing microfragments and plastic load was recorded.

## Supplementary Information

Supplementary Information.

## Data Availability

The datasets generated during and/or analyzed during the current study are available from the corresponding author on reasonable request.
