# Supplementary material for: Methods for controlled preparation and dosing of microplastic fragments in bioassays
Source: Sci Rep. 2023 Mar 30;13:5195. doi: 10.1038/s41598-023-32250-y (PMC10063547; doi:10.1038/s41598-023-32250-y)
Supplement: Supplementary file 1 — Supplementary Information. [file 41598_2023_32250_MOESM1_ESM.docx]

**Supplementary Information For**

**Methods For Controlled Preparation and Dosing of Microplastic Fragments in Bioassays**

**Hayden Boettcher^1,*^, Tobias Kukulka^2^, Jonathan H. Cohen^1^**

^1^ University of Delaware, School of Marine Science and Policy, Lewes DE, 19958, United States

^2^ University of Delaware, School of Marine Science and Policy, Newark DE, 19971, United States

^*^hayden@udel.edu

**Table S1. Summary statistics from the microfragment linear regressions.** **With respect to the equation variables, y= target number of microplastic fragments and x= mass of microplastics to be weighed out in mg.**

| **Polymer Type** | **Size Class** | **Equation** | ***p*-value** | **Correlation** | **Adj R-squared** |
| --- | --- | --- | --- | --- | --- |
| Polyethylene | 53-150 μm | y= (4604*x) | < 9.647e-14 | 0.9377208 | 0.9464 |
|  | 150-300 μm | y= (632*x) | < 2.2e-16 | 0.9953707 | 0.995 |
|  | 300-1000 μm | y= (48.43*x) | < 7.986e-12 | 0.9584952 | 0.9564 |
| Polypropylene | 53-150 μm | y= (5101*x) | < 3.211e-14 | 0.9709348 | 0.9732 |
|  | 150-300 μm | y= (611.4*x) | < 6.934e-14 | 0.9782348 | 0.9768 |
|  | 300-1000 μm | y= (83.77*x) | < 2.2e-16 | 0.9910787 | 0.9907 |
